# Identifications of conserved 7-mers in 3'-UTRs and microRNAs in *Drosophila*

**DOI:** 10.1186/1471-2105-8-432

**Published:** 2007-11-08

**Authors:** Jin Gu, Hu Fu, Xuegong Zhang, Yanda Li

**Affiliations:** 1 Bioinformatics Division, TNLIST and Department of Automation, Tsinghua University, Beijing 100084, China

## Abstract

**Background:**

MicroRNAs (miRNAs) are a class of endogenous regulatory small RNAs which play an important role in posttranscriptional regulations by targeting mRNAs for cleavage or translational repression. The base-pairing between the 5'-end of miRNA and the target mRNA 3'-UTRs is essential for the miRNA:mRNA recognition. Recent studies show that many seed matches in 3'-UTRs, which are fully complementary to miRNA 5'-ends, are highly conserved. Based on these features, a two-stage strategy can be implemented to achieve the *de novo *identification of miRNAs by requiring the complete base-pairing between the 5'-end of miRNA candidates and the potential seed matches in 3'-UTRs.

**Results:**

We presented a new method, which combined multiple pairwise conservation information, to identify the frequently-occurred and conserved 7-mers in 3'-UTRs. A pairwise conservation score (PCS) was introduced to describe the conservation of all 7-mers in 3'-UTRs between any two *Drosophila *species. Using PCSs computed from 6 pairs of flies, we developed a support vector machine (SVM) classifier ensemble, named Cons-SVM and identified 689 conserved 7-mers including 63 seed matches covering 32 out of 38 known miRNA families in the reference dataset. In the second stage, we searched for 90 nt conserved stem-loop regions containing the complementary sequences to the identified 7-mers and used the previously published miRNA prediction software to analyze these stem-loops. We predicted 47 miRNA candidates in the genome-wide screen.

**Conclusion:**

Cons-SVM takes advantage of the independent evolutionary information from the 6 pairs of flies and shows high sensitivity in identifying seed matches in 3'-UTRs. Combining the multiple pairwise conservation information by the machine learning approach, we finally identified 47 miRNA candidates in *D. melanogaster*.

## Background

MiRNAs are a class of ~22 nt endogenous small RNAs which regulate target mRNAs by repressing the translation or directly degrading the mRNA transcripts [1,2]. MiRNAs take part in several essential biological processes, such as development, metabolism, cell differentiation and aging [1,2]. To date, 78 miRNAs and few miRNA:mRNA interactions have been experimentally identified in *Drosophila *[3,4]. In an early computational study, Lai et al. estimated that the fly genome contains around 110 miRNA genes [5]. Applying the high-throughput pyrosequencing method on mixed-stage *C. elegans*, Ruby et al. confidently identified 112 miRNAs while missing 19 annotated [6]. *C. elegans *genome may contain around 150 miRNAs. Although the number of protein-coding genes in a fly (14,000) is less than in a worm (18,000), the number of body cells in a fly is ten times more than a worm. The total number of miRNAs in a fly can also be expected to be around 150, which is similar to a worm. These studies suggest that another 40~70 miRNAs still need to be discovered.

Many miRNA prediction [5,7-14] and target prediction algorithms [15-19] have been introduced in recent years. But most of the previous studies took miRNA prediction and target prediction as two separate tasks. The functions of the predicted miRNAs are hard to be explored because of inaccurate prediction of the 5'-ends of mature miRNAs. In a recent study, Nam et al. reported that the mean distances between their predicted 5'-ends of mature miRNAs and the experimental identified 5'-ends are about 2 nt (nucleotide) [10]. Several studies showed that the base-pairing between the 5'-end of the mature miRNA and the target mRNA 3'-UTRs is essential for the miRNA:mRNA recognition and the 7 or 8 nt miRNA seed matches (the 7 or 8 nt sequences fully complementary to the 5'-ends of miRNA in the 3'-UTRs) are highly conserved in 3'-UTRs [20-22]. Based on these features, a new strategy combining the prediction of miRNA and their target prediction was introduced: first, they identified conserved motifs in 3'-UTRs; second, they regarded these conserved motifs as candidate seed matches derived from miRNA binding sites and then used them to search for complementary sites in the genome; finally, two ~100 nt sequences were extracted according to each matched locus and miRNAs were predicted from these ~100 nt sequences [23,24].

Comparative genomic methods are useful to identify conserved sequence motifs [23-30]. Most of studies only focus on motifs in the promoter regions. Seed matches corresponding to miRNA binding sites in 3'-UTRs have several features: 1) the length of conserved sites is 7 or 8 nt; 2) tens of different mRNAs contain a same seed match, and may be regulated by the same miRNA; 3) many seed matches are highly conserved in 3'-UTRs [15,16,20-22]. Xie et al. and Chan et al. presented different algorithms to analyze "common regulatory motifs" in 3'-UTRs. Xie et al. presented a motif conservation score (MCS) to identify frequently-occurred and conserved motifs in 3'-UTRs from 4-way alignments of mammals and predicted 207 human miRNAs based on the identified motifs in 3'-UTRs [23]. The MCS scoring method only considers the conservation ratio of motifs. The motifs with small counts may have pseudo higher or lower conservation ratios during the evolutions (according to the law of large numbers). These motifs will produce noises when identifying conserved motifs. Chan et al. used a non-alignment based method (FastCompare) to identify conserved k-mers in worm and fly [24,31]. This method can avoid the problem of misaligning homologous sequences. But this method needs a set of known homologous genes to start the analysis. Many latest sequenced genomes do not have accurate annotations of gene regions. For example, in *Drosophila*, at this time, nine genomes have been sequenced, but only *D. melangoster *and *D. pseudoobscura *gene annotations are available.

In this work, we presented a new scoring system and a pattern recognition method, which can identify "conserved motifs" which have high conservation ratio and frequent occurrences in aligned 3'-UTRs. We introduced a pairwise conservation score (PCS) to evaluate the "conservation" of 16,384 7-mers independently in the 3'-UTRs of 6 pairs of flies. Then we developed a support vector machine (SVM) ensemble, named as Cons-SVM, to identify conserved 7-mers having similar conservation patterns with the reference seed matches along the phyla. We identified 689 conserved 7-mers including 65 out of 86 reference seed matches (seed matches, the 7-mers complementary to the 1–7 nt and 2–8 nt of mature miRNAs). Following study showed that Cons-SVM has higher sensitivity than previous methods for identifying seed-match-like conserved 7-mers.

The second stage of our work was to identify miRNA candidates based on the 689 conserved 7-mers. Introducing the seed match information into released miRNA prediction methods can increase the specificity and can also more accurately predict the 5'-ends of mature parts on the predicted pre-miRNA candidates. Different to previous studies using the similar strategy, we designed a more detail method to identify pre-miRNA candidates and the corresponding mature parts. We first explored whether the 90 nt flanking sequences having the complementary sites to any conserved 7-mer in the whole genome can form conserved stem-loops. Using the miRNA prediction method RNAmicro [14], we identified 97 pre-miRNAs including 41 new pre-miRNA candidates not collected in miRBase. Then, we introduced several rules to annotate the mature parts in the predicted pre-miRNA candidates. 47 mature miRNA candidates are identified on the 41 pre-miRNA candidates. Eight/seven of them can find homologies in mosquito/honeybee. Then we predicted the target genes of any miRNA candidate simply by investigating whether the 3'-UTRs of specific genes have one or more conserved seed matches of that candidate.

The two-stage method successfully identified many new miRNA candidate and their binding sites in 3'-UTRs, revealing extensive miRNA:mRNA interactions in fly.

## Results and Discussions

**Figure 1:**
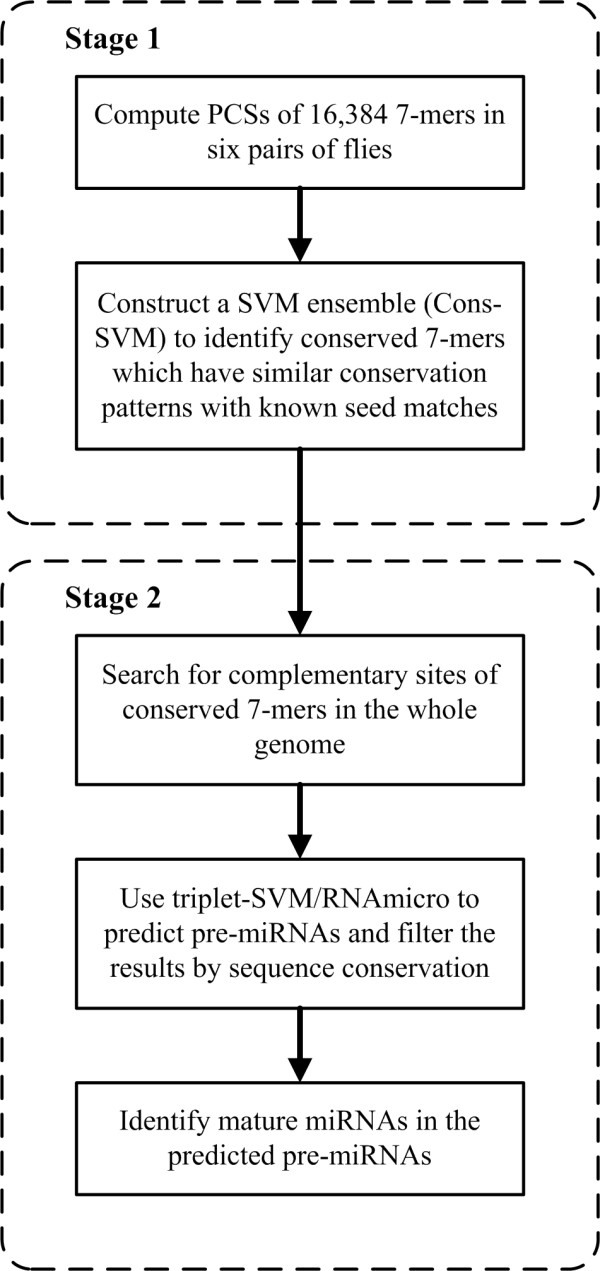
**The flowchart of the method**. The whole method consists of two stages: in the first stage, conserved 7-mers are identified by considering all 7-mers' conservation patterns in six pairs of flies; in the second stage, pre-miRNAs and mature miRNAs are predicted by adding seed-matching information into published miRNA prediction methods in the whole genome.

We used a two-stage method to identify conserved 7-mers in 3'-UTRs and miRNA:mRNA interactions in *Drosophila *(Figure 1). In the first stage, conserved 7-mers were identified by considering the multiple pairwise conservations of 16,384 (4^7 ^= 16,384) 7-mers in seven flies' 3'-UTRs. In the second stage, the conserved 7-mers were used to search for pre-miRNA candidates in the whole genome. Then the 5'-ends of mature miRNA candidates were annotated based on sequence features. Finally, the target genes of the miRNA candidates were analyzed.

### The reference dataset

In this work, seven flies were studied (the abbreviated and full names of the studied organisms: *D. melanogaster*, Dme; *D. simulans*, Dsi; *D. yakuba*, Dya; *D. ananassae*, Dan; *D. pseudoobscura*, Dps; *D. mojavensis*, Dmo; *D. virilis*, Dvi). For the 78 mature miRNAs collected in miRBase, 59 miRNAs are identified by cloning, 16 are only verified by northern blotting and the other 3 are predicted by sequence homologies [3-5]. The 5'-ends of the 59 miRNAs identified by cloning (corresponding to 61 unique pre-miRNAs) are accurately determined, so we used them as the references (Table S1, Additional file 4). We extracted a set of 86 non-redundant seed sequences according to the 1–7 nt and 2–8 nt of the 59 miRNAs. The 59 miRNAs can be clustered into 40 unique families based on their seed sequence similarities. The 86 non-redundant seed matches fully complementary to miRNA seed sequences were used as positive samples in the following analysis.

### The conservation ratio and the count of seed matches in 3'-UTRs

To investigate the two "variables" of seed matches, we compared the conservation ratios (conservation ratio for a 7-mer is defined as its count in the conserved regions divided by the count in the original sequences) and the number of occurrences in 3'-UTRs among three defined datasets: the "seed matches" dataset containing the 86 non-redundant reference seed matches, the "shuffled seed matches" dataset (having the same nucleotide content as the seed matches dataset, every seed match was shuffled 5 times), and the "all 7-mers" dataset.

Seed matches tend to be conserved in 3'-UTRs [15,16,20-22]. In our study, we also found that the conservation ratios for seed matches are much larger than those for the other 7-mers. Requiring Dme-Dps pairwise conservation, the mean of the conservation ratios of the 7-mers in the "seed matches" (0.2816) dataset is significantly higher than that in the "shuffled seed matches" dataset (0.1160, p-value: 0) and in the "all 7-mers" dataset (0.1151, p-value: 0). Tens of different mRNAs contain the same seed match, and may be regulated by the same miRNA [15,16,21]. We wondered whether seed matches have more occurrences than other 7-mers. In the original Dme 3'-UTRs, the mean of the count of 7-mers in the "seed matches" dataset (278.7) is weakly higher than that in the "shuffled seed matches" dataset (257.9, p-value: 0.2455) and in the "all 7-mers" dataset (236.9, p-value: 9.4118e-004). These results suggest that the higher conservation ratios is an effective feature to identify seed-match-like 7-mers in 3'-UTRs, while the more occurrences may help identify the seed matches with excessive counts but reduce the sensitivity for the seed matches with depletive counts (The histograms of the conservation ratios and the number of occurrences are shown in Figure S1, Additional file 1). Wilcoxon rank sum test was used to test the mean difference.

### Computation of pairwise conservation scores for all 7-mers

We introduced a pairwise conservation score (PCS), which is defined as the log rank ratio between the counts in the original Dme 3'-UTRs and in the pairwise-conserved 3'-UTRs, to evaluate the "conservation" of each 7-mer in a pair of flies (see detail in Methods). Zero PCS means that a 7-mer is under neutral evolution and larger PCS means that a 7-mer is more conserved. The PCS favours the 7-mer with higher conservation ratio and also weakly prefers more occurrences. Take a reference miR-12 as the example. The 7-mer seed match complementary to the 1–7 nt of miR-12, "ATACTCA", has 292 occurrences in Dme, with 71 conserved in the Dme-Dps pair. However, a non-seed-match 7-mer, "ATACTTG", has 287 occurrences in Dme, but only 26 conserved in the Dme-Dps pair. Another non-seed-match 7-mer, "GTAGGCC", has similar 24.6% (15/61) occurrences conserved, but only 15 occurrences can be found in the Dme-Dps pair. The PCS of the seed match "ATACTCA" is 0.889, larger than the PCSs of "ACGTCAC" and "GTAGGCC" -0.424 and 0.498, respectively.

3'-UTRs are highly AU-rich (in the studied 3'-UTR set, AU-content 62.6%). Different AU-contents of different 7-mers may bias their corresponding PCSs. We compared the mean value (in Dme-Dps pair) and the distribution of PCSs among the three datasets defined in the previous section. The PCSs of the "seed matches" dataset have significantly higher mean value (0.97) than that of the "shuffled seed matches" dataset (-0.042) and the "all 7-mers" dataset (8.3e-006). While the distribution of the PCSs of the "shuffled seed matches" dataset shows no significant difference with that of the "all 7-mers" dataset (p value: 0.1262). The near zero mean value of the PCSs of the "shuffled seed matches" dataset and the similar distribution of the PCSs between the "shuffled seed matches" dataset and the "all 7-mers" dataset suggest that the shuffled seed matches have similar PCSs as the background (all 7-mers). In summary, the PCSs of the "seed matches" dataset differentiate significantly with those of the "all 7-mers" dataset (background), but the PCSs of the "shuffled seed matches" dataset, having the same nucleotide-content with the "seed matches" dataset, show no significant difference with the "all 7-mers" dataset (background). This result indicates that the nucleotide content does not bias the PCSs of different 7-mers. Wilcoxon rank sum test was used to test the mean difference, and two-sample Kolmogorov-Smirnov goodness-of-fit hypothesis test was used to test the distribution difference.

For all 16,384 (4^7^) 7-mers, we computed their PCSs in 6 pairs of flies (Dme-Dsi, Dme-Dya, Dme-Dan, Dme-Dps, Dme-Dmo and Dme-Dvi pairs). The histograms and tables of PCSs (Figure S2, Additional file 2) and conservation ratios (Figure S3, Additional file 3) were show in Table S2 (Additional file 5). In the Dme-Dsi pair, the distribution of the PCSs of the 86 reference seed matches is indistinguishable from that of all 7-mers and located around 0, although the PCSs of the seed matches tend to be larger than 0. As the evolution distance increases, the PCSs of the seed matches disperse gradually. The numbers of seed matches scoring higher than 0 are 75, 78, 80, 80, 80 and 79 in the six pairs of flies, and 80 for the average score.

### Identification of "conserved" 7-mers by Cons-SVM

To identify "conserved" 7-mers having similar conservation patterns with seed matches, we combined the 6 PCSs, which characterize the conservation pattern of each 7-mer in the *Drosophila *phyla, to form a feature vector and then we developed a SVM classifier ensemble to identify the 7-mers having the similar conservation pattern with the 86 reference seed matches.

We used all the PCSs of each 7-mers in 6 different pairs of flies as the features to describe their conservations in the seven studied flies. The 86 seed matches derived from the 59 reference miRNAs were used as positive training samples. Another 86 7-mers randomly sampled from all the other 7-mers were used as negative training samples. The SVM classifier was trained based on these two sample sets. Then the trained SVM was used to classify all the 16,384 7-mers into conserved 7-mers and non-conserved ones. To control the variations of the randomly sampling for the negative samples, we repeated the sampling 500 times and trained 500 SVMs. The outputs of the 500 SVMs were combined as a classifier ensemble by a voting strategy. To reduce false positives, we used a stringent voting strategy that a sample was classified as positive only if it was classified as positive in all 500 SVMs. We call the classifier ensemble as Cons-SVM.

Applying Cons-SVM on all the 16,384 7-mers, 689 of them were classified as positive, including 65 reference seed matches. To estimate the false positives of identifying "conserved" 7-mers, we repeated the same approach on the random dataset: PCSs were computed from the random 3'-UTRs, and then the same Cons-SVM was applied on the combined PCS vectors. All the samples derived from the random dataset should be negative samples. So we regarded the identified 56 7-mers derived from the random dataset as false positives. The false positive rate was estimated as 8.1% (56/689). We then used a cross validation method (see detail in Methods section) to test the sensitivity of the Cons-SVM. 63 out of 86 seed matches were identified as positives (Sensitivity 73.3%). These 63 seed matches could match 52 out of 59 reference miRNAs (33 out of 40 miRNA families, sensitivity 82.5%). Cons-SVM has achieved high sensitivity for identifying "conserved" 7-mers with similar conservation patterns of reference seed matches in 3'-UTRs, but a few real seed matches with weaker conservation and lower count will be missed.

### Comparisons with other methods

We next compared the results with the MCS algorithm and the FastCompare algorithm [23,24]. The MCS algorithm quantified the extent of excess conservation of a motif by considering that the observed conservation rate of the motif exceeds the conservation rate for comparable random motifs in multiple alignments. The FastCompare algorithm used network-level conservation [31] to evaluate the conservation of k-mers in pairs of species.

We used the 689 highest scoring 7-mers from the other two methods to compare with our results of Cons-SVM. Results show that Cons-SVM has higher sensitivity than FastCompare and the MCS algorithm. Because only Dme-Dps conservation information was used in the FastCompare algorithm, we also compared the performance of the three algorithms under the same condition (we used PCSs computed only in Dme-Dps pair instead of Cons-SVM). Results show that the PCS scoring method also shows higher sensitivity than the other two algorithms (Table 1). The specificities were not compared because these algorithms needed different strategies to produce randomized data: in our work we randomized the Dme 3'-UTRs and 6 pairwise alignments, for the MCS algorithm we should randomize the multiple alignments and for the FastCompare we should randomize the 3'-UTRs of the two studied species.

**Table 1:** Performance comparisons of different algorithms

The organisms selected for analysis	The algorithm	The number of Identified reference seed matches^1^
Dme Dsi Dya Dan Dps	Cons-SVM	63^2 ^(33^3^)
Dmo Dvi		
Dme Dsi Dya Dan Dps	MCS [23]	58(29)
Dmo Dvi		
Dme Dps	FastCompare [24]	52(29)
Dme Dps	PCS^4^	59(32)
Dme Dps	MCS [23]	47(26)

^1 ^Because Cons-SVM identifies 689 candidate seed matches, we test the performances of different algorithms when selecting the 689 highest ranking 7-mers.

^2 ^This number is obtained by LOOCV (Leave One Out Cross Validation). The number of identified reference seed matches is 65 when classification.

^3 ^The numbers in the parenthesis indicate how many miRNA families are identified according to the identified seed matches.

^4 ^We only used the PCSs computed from Dme-Dps pairs and used the 689 highest-score 7-mers in the analysis.

**Figure 2:**
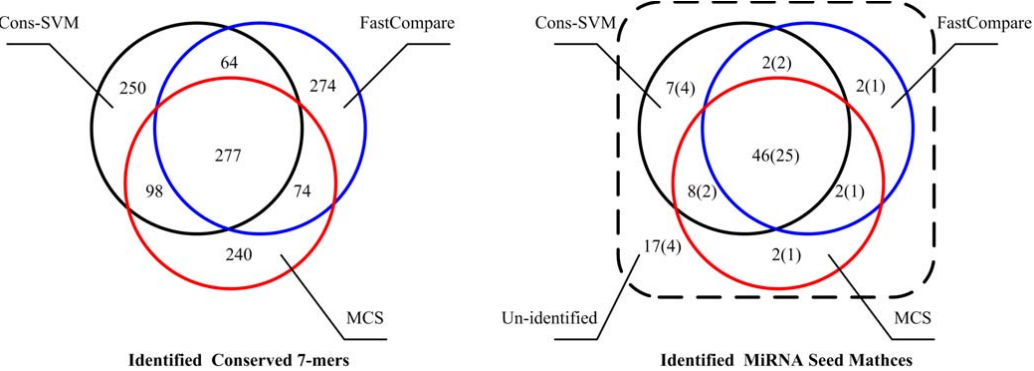
**Comparions of the results using the three methods**. The number in each block indicates the corresponding number of 7-mers in that part. The number in the parenthesis indicates the number of reference miRNA families in that block.

For the 689 high-scoring 7-mers, 277 are identified by all three methods, 236 are identified by only two different methods, and 764 7-mers are identified by only one method. This result suggests that the three methods extract different information from the genomic data and new experimental data are needed to evaluate the accuracy of the three methods. But the result is much more consistent for identifying miRNA seed matches, 46 seed matches (25 families) are identified by all three methods, 12 (5 families) are identified by only two different methods, and 11 (6 families) are identified by only one method (Figure 2). Tabulated details for each reference miRNA are presented in Table S3 (Additional file 6).

### Prediction of pre-miRNAs

3'-UTRs contain many other conserved regulatory elements except miRNA seed matches. The AU-rich elements (UAAUUUA, UUAUUUA), the proneural box (aauggaAGACAAU), and the alcohol dehydrogenase 3'-UTR downregulation control element (AAGGCUGa) can also be found in the 689 identified conserved 7-mers. What remains to be answered is how many conserved 7-mers are potentially miRNA target sites. We implemented genome-wide miRNA predictions using two published miRNA prediction methods while introducing one additional feature: whether the predicted miRNAs have at least one conserved site complementary to one of the identified 689 conserved 7-mers.

All the 689 identified conserved 7-mers were searched in Dme's genome in both strands excluding all annotated exons, tRNAs, snRNAs, rRNAs and other noncoding gene regions. In each matched locus, two 90 nt sequences were extracted, one from -15 nt to +75 nt and the other from -55 nt to +35 nt. We filtered these sequences with free energy and basic stem-loop structural features (see details in Methods) and then predicted pre-miRNA candidates by two miRNA prediction methods triplet-SVM and RNAmicro. The two methods are chosen, because triplet-SVM shows higher sensitivity while RNAmicro has higher specificity [12,14]. Then we analyzed whether these predicted pre-miRNAs loci were conserved in each pair of flies: the pairwise alignments of each pre-miRNA were extracted from whole-genome pairwise alignments of each pair of flies (the data downloaded from the UCSC Genome Browser ftp site); a predicted pre-miRNA locus was regarded as conserved between the two flies, if 1) the corresponding regions are aligned in the UCSC pairwise alignments, 2) the "seed" sequences (the 7-nt fully complementary to any conserved 7-mer) was totally identical [7], and 3) the aligned sequence of the second organism was also predicted as a pre-miRNA by the miRNA prediction method. A predicted pre-miRNA locus was taken for following analysis, if it was conserved in at least four pairs of flies. Then the pre-miRNA candidates overlapped in their genome locations were clustered into a single miRNA locus. The locus with the minimal free energy was selected as the representative pre-miRNA candidate of the cluster. Due to limited space, here we only presented the results when we used the miRNA prediction method RNAmicro. The results using triplet-SVM is reported via our website [45].

According to the above steps, we identified 97 pre-miRNA candidates (using RNAmicro) including 46 pre-miRNAs in the 61 reference pre-miRNAs (Additional file 6). In the 15 missed reference pre-miRNAs, 4 did not pass the pre-processing filter due to their predicted double-loop structures (mir-2c, mir-31a, mir-31b, mir-286) and another 2 due to their low predicted free energy (mir-309, mir-311). So the sensitivity on the reference set should be 83.6% (46/55). Another set of 7 pre-miRNAs collected by miRBase are also identified. For the remaining 44 predicted pre-miRNAs, 3 are mapped to the minus strand of reference pre-miRNAs (mir-5, mir-9c, mir-iab-4), and the other 41 are new pre-miRNA candidates which we named as "dme-pmir-1" to "dme-pmir-41" (Additional file 7). Three pre-miRNAs candidates are located in alternative regions of protein-coding genes: pmir-29 (intron:-:Brf-RA|exon:+:CG5319-RA), pmir-13 (exon:+:Glycogenin-RB|intron:+:Glycogenin-RA) and pmir-18 (intron:-:CG9238-RA|exon:-:CG9238-RB). In human, mir-17~92-2 cluster is located in an alternative region of a protein-coding gene and the miRNAs in the cluster may be related to cancers [32,33]. So we kept the three predictions.

Lai et al. reported the 210 top scoring pre-miRNA candidates using the miRSeeker pipeline [5]. They identified 47 reference pre-miRNAs and a set of 9 pre-miRNAs also collected in miRBase, in which 40 and 4 are identified by our method, respectively. For their remaining predictions, 15 candidate miRNAs can also be predicted by our method. Chan et al. predicted 92 pre-miRNAs in their work [24]. They only predicted 12 reference pre-miRNAs. And for the remaining 80 new predictions, 10 are overlapped with Lai et al. and 4 with our method (pmir-26-5, pmi-24a; pmir-9-5, pmi-287a; pmir-16-5, pmi-238a; pmir-5-3, pmi-148c). Only 2 predictions are reported in all the three methods (rank 24, pmir-16-5, pmi-238a; rank 57, pmir-5-3, pmi-148c). The results indicate that our method for identifying miRNAs has high sensitivity, but the specificity remains unclear due to limited consistency of the predictions.

### Identifications of mature miRNAs

**Figure 3:**
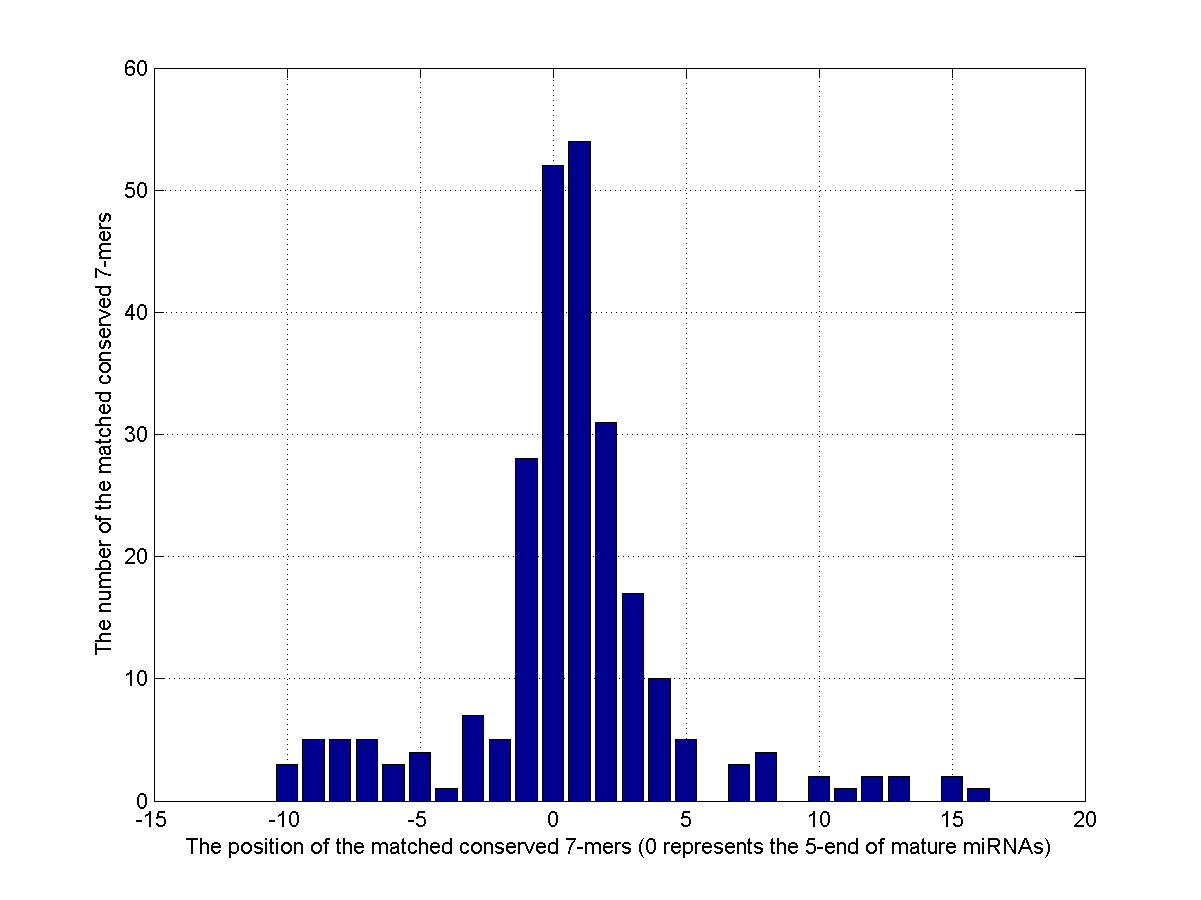
**The 689 conserved 7-mers identified by Cons-SVM matching with the 59 reference miRNAs**. Much more sites matched with the 1–7 nt or 2–8 nt of the mature miRNAs.

**Figure 4:**
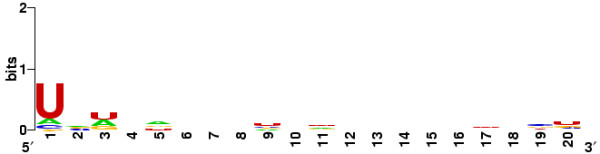
**The nucleotide composition of the 59 reference miRNAs**. The 5' first nucleotide of mature miRNAs significantly favours "U". Other sites do not show similar nucleotide bias. The logo plot is produced by WebLogo [49].

Next, we annotated the mature parts on the two arms of the predicted pre-miRNAs. We observed that the conserved 7-mers matched to the 1–7 nt and 2–8 nt of known mature miRNAs were significantly more than those matched to other loci (Figure 3). We also observed that the first nucleotide of mature miRNAs favoured "U" (Figure 4). This phenomenon was reported in an early study in *C. elegans *[34]. Based on these two observations, we introduced several rules to identify the 5'-end of mature parts on the two arms of the predicted pre-miRNA candidates. We investigated the number of conserved sites complementary to the conserved 7-mers and whether these conserved sites having "U" as the first nucleotide (see details in Methods).

The identified 46 reference pre-miRNAs contain 43 unique reference mature miRNAs (33 families). Following the rules presented in Methods, we correctly predicted the 5'-ends (first or the second nucleotide) of 33 mature miRNAs (27 families), with accuracy 76.7% (33/43) (Table S3, Additional file 6). In the 33 correct predictions, we retrieve 29 exact 5'-ends (23 families) and the 4 off by +1 nt. MiR-133, miR-219, miR-263a, miR-274, miR-281-2*, miR-282, miR-283 and miR-310 which are also collected in miRBase without cloning evidence, are also identified. But the predicted 5'-ends of miR-263a, miR-274, miR-282 and miR-283 are very different with current annotations. We predicted that they should start at the 6th, 3rd, 4th and 3rd nucleotide of the current annotated mature sequences, respectively. The four miRNAs are computationally predicted and validated by northern blot [5]. The sequence lengths of the four miRNAs are much longer than other miRNAs (24, 26, 28 and 21 nt long, respectively). The results suggest that the accurate 5'-ends of these miRNAs should be further validated. Two pre-miRNAs only identified mature miRNAs on the star (*) arm (mir-10, mir-285). And ten pre-miRNAs also predicted mature parts on the star (*) arm (mir-305, mir-79, let-7, mir-2a-2, mir-8, mir-7, mir-9a, mir-316, mir-34, mir-12).

Using RNAmicro software, we identified 41 pre-miRNA candidates. And then we predicted 47 mature miRNA candidates on these candidates. Three of the mature candidates have sequence homologies to known miRNAs in other species and 8/7 can find homologies in mosquito/honeybee's genome (Table 2). Detail information for all predicted pre-miRNA candidates and their corresponding mature parts is all presented in Table S4 (Additional file 7).

**Table 2:** The list of predicted miRNAs which have homologies with other known miRNAs or conserved in other insects

**MiRNA**	**Mature Sequnece**	**Genomic location**	**Other**	**Ag**	**Am**
pmir-1	TAAGCGTAtagcttttcccct	chr2L:Minus:Intron 243041–243130	Rank#197^a^	+	+
pmir-11	TTATTGCTtgagaatacacgt	chr2R:Minus:Intergenic 11580118–11580207	tni-miR-137 Rank#55	+	+
pmir-16	GATATGTttgatattcttggt	chr3L:Plus:Intron 8545755–8545844	cbr-miR-50 Rank#24 pmi-238a^b^	+	+
pmir-20	AATTGACTctagtagggagtc	chr3R:Plus:Intron 121093–121182	Rank#5	+	+
pmir-26	TAAGTACtagtgccgcaggag	chr3R:Minus:Intron 9289943–9290032	cel-mir-252 cbr-mir-252 pmi-24a	+	
pmir-29	ATGCAACgttgctgggaagtg	chr3R:Plus:Intron 13213562–13213651			+
pmir-31	TGTTAACtgtaagactgtgtc	chr3R:Minus:Intron 17623957–17624046		+	
pmir-33	TATTGTCCtgtcacagcagta	chr3R:Minus:Intergenic 21414590–21414679	Rank#119	+	+
pmir-37	TTCGTTGTcgacgaaacctgc	chrX:Minus: Intergenic 1645018–1645107	Rank#15	+	+

^a ^The miRNA candidates also predicted by Lai et al [5].

^b ^The miRNA candidates also predicted by Chan et al [24].

### Analysis of miRNA targets

We predicted the target genes of the 47 predicted mature miRNAs candidates simply by investigating whether the conserved regions (conserved in Dme-Dps pair) of the 3'-UTRs of specific genes contains one or more seed matches of each miRNA. Then we used GeneMerge to analyze the function enrichments of the target genes for each miRNA. GeneMerge is a program which can provide statistical rank scores for over-representation of particular GO categories [36-38] for a given set of genes [35]. Significant functional categories (Bonferroni corrected p-value < 0.001) are reported in Table 3. The target genes of 5 miRNA candidates are enriched in transcriptional activity (pmiR-7-5, 8-5, 10-3, 15-5, 32-5), and 2 are enriched in protein binding (pmiR-3-5, 25-5).

**Table 3:** The list of predicted miRNAs which have significant GO categories (with Bonferroni corrected P-value less than 0.001)

**MiRNA**	**GO Category Description**	**P-value**
pmiR-3-5	protein binding	1.59E-03
pmiR-5-3	receptor activity	8.77E-03
	cell adhesion molecule binding	1.45E-04
pmiR-7-5	transcription factor activity	5.07E-03
pmiR-8-5	transcription factor activity	2.70E-03
	specific RNA polymerase II transcription factor activity	1.61E-03
	structural constituent of cytoskeleton	7.38E-04
pmiR-10-3	DNA binding	9.60E-03
	SH3 domain binding	3.19E-03
	specific RNA polymerase II transcription factor activity	2.14E-03
pmiR-13-3	cell adhesion molecule binding	1.78E-03
pmiR-15-5	transcription factor activity	4.73E-07
	RNA polymerase II transcription factor activity	6.85E-07
	protein serine/threonine kinase activity	6.71E-05
pmiR-24-3	DNA binding	2.93E-03
	structural constituent of cytoskeleton	8.08E-03
pmiR-25-5	protein binding	6.78E-03
pmiR-28-3	guanyl-nucleotide exchange factor activity	7.01E-03
pmiR-31-3	potassium channel activity	7.01E-03
pmiR-32-5	specific RNA polymerase II transcription factor activity	3.75E-04
pmiR-36-5	phosphatidylcholine-sterol O-acyltransferase activity	1.61E-03
pmiR-39-5	receptor binding	5.22E-03

We also analyzed the target and anti-target gene groups with the same GO categories [39]. We calculated the significance of seed enrichment in specific groups of genes for three datasets: the 59 reference miRNAs, the 47 new miRNAs candidates and the 9 candidates with additional conservation in mosquito or honeybee (see detail in Method). We curiously found that the target and anti-target groups are not consistent within the reference miRNAs and the new candidates. Many GO categories enriched for seed matches of the reference miRNAs (target groups), such as *nervous system development*, *regulation of transcription from RNA polymerase II promoter *and *DNA binding*, are not enriched for the seed matches of the new candidates. *Eye development *(corrected p-value: 0.0022143) and *integral to membrane *(corrected p-value: 0.05618) are the two top target GO categories of the new candidates. For anti-target GO categories, *structural constituent of ribosome *genes avoid both the seed matches of the reference miRNAs and the new candidates; *DNA binding *(corrected p-value: 0.045038) and *specific RNA polymerase II transcription factor activity *(corrected p-value: 0.089004) genes even significantly avoid the seed matches of the 9 ultra-conserved candidates. This difference is an interesting problem and still needs further study to answer it. Detail results are presented in Table S5 (Additional file 8).

## Conclusion

To reveal miRNA-directed posttranscriptional regulations in *Drosophila*, we used a two-stage method. We first used the conservation pattern along the phyla to identify conserved 7-mers. A pairwise conservation score (PCS) was introduced to describe the pairwise conservation of all 7-mers. Then a SVM ensemble was developed to combine the PCSs in 6 different pairs of flies. We identified 689 conserved 7-mers in the first stage. In the second stage, we tried to identify the candidate seed matches potentially involved in miRNA regulations and their corresponding miRNAs. We used all the identified 7-mers to search for pre-miRNAs. Then we manually annotated predicted miRNA genes and the 5'-ends of mature miRNAs according to conservation and sequence information. Finally, we identified 47 miRNA candidates. Target genes of each miRNA candidate were analyzed. Results show that many target and anti-target GO categories are different between the known miRNAs and the new predictions.

## Methods

### The sequences of the genomes and the 3'-UTRs

The genomes of *D. melanogaster *(dm2), the pairwise alignments of 6 pairs of flies (Dme-Dsi, Dme-Dya, Dme-Dan, Dme-Dps, Dme-Dmo and Dme-Dvi; following genome assemblies were used to construct the alignments: dm2, droSim1, droYak, droAna, dp3, droMoj1 and droVir1) were downloaded from UCSC Genome Browser ftp site [40,41]. The Dme 3'-UTRs were extracted from the genome sequences according to flybase 3'-UTR annotations (version 4.2.1). The pairwise alignments of 3'-UTRs were extracted from the whole genome pairwise alignments also according to the flybase annotations. If multiple 3'-UTRs existed in a single gene, the 3'-UTRs were merged as one sequence with the maximum coverage. We finally constructed a 3'-UTRs dataset containing 9,803 fly genes.

The mosquito (anoGam1) and honeybee (apiMel2) genomes were also downloaded from UCSC Genome Browser ftp site.

The Dme 3'-UTRs were randomized using python scripts written by Peter Clote for the Altschul-Erikson algorithm [42]. The pairwise alignments of 3'-UTRs were randomized using Perl scripts written by Stefan Washietl [43].

### The sequences of miRNAs

The sequences 78 pre-miRNAs and 78 mature miRNAs were downloaded from miRBase (Version 8.0) [44]. In the 78 mature miRNAs, 59 are identified by cloning, 16 are computational predicted and validated by northern blotting, and the other 3 are verified by distant homologies. The list of the 59 cloning-identified miRNAs can be found in Table S3 (Additional file 6).

The 59 cloning-identified miRNAs and corresponding 61 unique pre-miRNAs were used as the reference dataset in this work. The seed matches of each of the miRNAs were derived from the full complementary sequences to 1–7 nt and 2–8 nt of the 59 miRNAs. The seed matches with the same sequences were only considered once.

### Pairwise conservation score (PCS)

The pairwise conservation score (PCS) is defined as follows,



r_k0 _is the rank of the number of the occurrences of the studied 7-mer in Dme 3'-UTRs, and r_ki _is the rank of the number of the occurrences of the studied 7-mer in the studied pairwise alignments of 3'-UTRs. Larger PCS for a k-mer means that larger portion and more number of the k-mer sites are left after evolution. The Perl script to compute PCSs is available for free download via our website [45].

### Cons-SVM

We used the bagging method [46] to alleviate the variations caused by the unbalance of the number of positive and negative samples. We used the 86 reference seed matches as positive training samples and randomly sampled 86 from the other 7-mers as negative training samples to train a SVM. The procedure was repeated 500 times. Then all 500 SVMs were combined as an ensemble. Any sample which was classified as positive in all 500 SVMs was regarded as positive. We used LibSVM package [47] for all the analysis. Linear kernel with the default parameter was used to train each SVM.

We used the leave one out cross validation method (LOOCV) to test the sensitivity of Cons-SVM. The seed matches in one of the 40 miRNA families were selected as the testing samples in each time. The seed matches in the other 39 families were used as the positive training samples to train a new Cons-SVM following the above procedures. Then the new trained Cons-SVM was used to classify the testing samples. The total number of seed matches that were classified as positive was regarded as the final result.

### Pre-miRNAs prediction

Several steps were implemented to predict pre-miRNAs: 1) all identified conserved 7-mers were searched in the Dme's genome in both strands excluding all annotated exons, tRNAs, snRNAs, rRNAs and other noncoding gene regions. 2) for each matched locus, two 90 nt sequences were extracted: one was from -15 to +74, and another one was from -54 to +35 (corresponding to the two potential pre-miRNAs, because mature miRNAs can either locate at the 5'-arm or the 3'-arm of the pre-miRNA). 3) these 90 nt sequences were folded by RNAfold [48], and those free energy higher than -25 kcal/m, more than one terminal loops, the base-pairs of the stem less than 20 bp, the distance from the matched 7-mer to the terminal loop less than 21 bp were filtered out (these filters are widely used in miRNA prediction algorithms); 4) candidate pre-miRNAs were predicted using triplet-SVM and RNAmicro [12,14]. Then the alignments of each candidate pre-miRNA were extracted from the pairwise alignments of the 6 pairs of flies. A pre-miRNA candidate was regarded as conserved in any pair of flies, if 1) the 7 nt fully complementary with any conserved 7-mers was totally identical, and 2) the aligned sequence in the second organism was also predicted as "real" pre-miRNAs by the miRNA prediction method. A pre-miRNA candidate which was conserved in at least 4 pairs of flies was regarded as a conserved pre-miRNA candidate. Then the conserved pre-miRNA candidates overlapped in their genome locations were clustered into one pre-miRNA locus, and the candidate having the lowest free energy of the predicted structure was denoted as the representative of the cluster.

### Mature miRNA prediction

We introduced several rules to identify the mature parts on the two arms of each predicted pre-miRNA: 1) if the predicted pre-miRNA only matched a single conserved site complementary to any conserved 7-mer, the conserved site complementary with the 7-mer was regarded as the 1–7 nt of the mature miRNAs. 2) if the predicted pre-miRNAs matched several conserved 7-mers, the 5'-most conserved site complementary to the conserved 7-mers had the first nucleotide as "U" was regarded as the 1–7 nt of mature miRNAs, if none of conserved site complementary to the conserved 7-mers had "U" as the first nucleotide, the 5'-most site was regarded as the 1–7 nt of mature miRNAs. The 21 nt sequence region from the predicted 5'-end of each mature miRNA is annotated as the candidate mature sequence.

All the predicted mature miRNA candidates were searched for homologies in miRBase, mosquito and honeybee genomes with BLAST program [50]. The hits with the length of aligned sequence longer than 19 nt and with maximal one mismatch were regarded as the homologies.

### Target Analysis

We first analyzed the enriched functional categories of target genes for each candidate miRNA. The target genes were simply predicted by searching for conserved 7-mers, which are complementary to the 5'-ends (1–7 nt and 2–8 nt) of mature miRNAs and in the 689 conserved 7-mers, in the aligned 3'-UTRs of specific genes in the Dme-Dps pair. Then we used GeneMerge [35] to analyze the GO categories of the target genes of each miRNA candidate.

Then we analyzed the target and anti-target groups of genes with the same GO categories. This analysis, proposed by Start et al., can be used to test whether the 3'UTRs in a functional category are specifically enriched for miRNA target sites over what is expected given their length [39]. First, we calculated the frequency of all 16,384 7-mers in all 3'-UTRs. We denoted the counts of seed match 7-mers and the all 7-mers in the Dme-Dps conserved 3'-UTRs as SeedM_Gene_All _and All_7M_Gene_All_, respectively. Then, we calculated the frequency of all 7-mers in 3'-UTRs of specific group genes (for example, the genes annotated as *central nervous system development*). We denoted the counts of seed match 7-mers and the all 7-mers in the 3'-UTRs of specific group of genes as SeedM_Gene_Specific _and All_7M_Gene_Specific_, respectively. Finally, we can assess the significance of seed enrichment for a group of genes by calculating the binomial probability (p value) that the observed level of enrichment is random, where the ratios for all genes define the background probability:



Bonferroni corrected p-value is also calculated.

## Authors' contributions

JG designed the algorithm, developed the PCS program and Cons-SVM program in Perl scripts and finished most of the manuscript. HF pre-processed the 3'-UTR dataset, developed the MCS algorithm, and re-developed the PCS program in C++. XZ provided useful guides for the experiment design and manuscript preparations. YL initiated the project and guided the whole work.

## Supplementary Material

Additional file 4 **The list of the 59 reference miRNAs identified by cloning**. Table S1. The list of the 59 reference miRNAs. All the entries are clustered according to miRNA family information. The number of homologies in six flies, the results of different motif finding methods and the results of mature miRNA identification are presented in the file. Click here for file

Additional file 1 **The distributions of conservation ratios and the counts in Dme-Dps pair**. Figure S1. The distributions are computed and plotted for three dataset: reference seed matches, shuffled seed matches and all 7-mers. A) The distribution of conservation ratios. B) The distribution of counts in Dme 3'-UTRs. C) The distribution of counts in Dme-Dps conserved 3'-UTRs. Click here for file

Additional file 2 **The histograms of PCSs and the trends of PCSs along the phyla**. Figure S2. A)-G) The PCSs of all 7-mers, from left to right: the average PCSs and the PCSs in Dme-Dsi, Dme-Dya, Dme-Dan, Dme-Dps, Dme-Dmo, Dme-Dvi. The top panel of each sub-figure shows the histograms of the PCSs of all the 7-mers and the bottom panel shows the enlarged visions. H) The trends of the PCSs of 86 seed matches along the phyla. Because the evolutionary distances of the Dme-Dmo and Dme-Dvi pairs are the same, only the PCSs of the Dme-Dmo pairs are displayed. Click here for file

Additional file 3 **The histograms of conservation ratios along the phyla**. Figure S3. A)-F) The conservation ratios of all 7-mers, from left to right: the conservation ratios in Dme-Dsi, Dme-Dya, Dme-Dan, Dme-Dps, Dme-Dmo, Dme-Dvi. The top panel of each sub-figure shows the histograms of the conservation ratios of all the 7-mers and the bottom panel shows the enlarged visions. Click here for file

Additional file 5 **The list of PCSs, counts and conservation ratios of all 7-mers**. Table S2. The list of the all 16,384 7-mers. The "Type" column describes whether a 7-mer is derived the 5'-end of cloned/northern blotting/homology miRNAs or predicted miRNA candidates. The "Class" column describes whether a 7-mer is classified as positive by Cons-SVM. The "PCS:XX" columns present the pairwise conservation scores in "XX" condition. The "BC:X" columns present the counts in single "X" 3'-UTRs. The "AC:XX" columns present the counts in "XX" conserved 3'-UTRs. The "CR:XX" columns present the conservation ratios in "XX" pair. Click here for file

Additional file 6 **The list of the 78 pre-miRNAs in miRBase and related annotations**. Table S3. The annotations of 78 pre-miRNAs and corresponding mature miRNAs. Click here for file

Additional file 7 **The list of the 41 predicted pre-miRNAs using RNAmicro**. Table S4. The annotations of 41 pre-miRNAs such as corresponding mature parts, genomic location, homologies, etc. Click here for file

Additional file 8 **The target and anti-target GO categories of miRNAs**. Table S5. The raw p-values and Bonferroni corrected p-values are reported for each GO category. The "Known_XX" columns are the p-values calculated for the 59 reference miRNAs. The "New_XX" columns are the p-values calculated for the 47 miRNA candidates. The "Cons_XX" columns are the p-values calculated for the 9 miRNA candidates with additional conservation in mosquito or honeybee. Click here for file

## References

[B1] Bartel DP (2004). MicroRNAs: genomics, biogenesis, mechanism, and function. Cell.

[B2] Ambros V (2004). The functions of animal microRNAs. Nature.

[B3] Lagos-Quintana M, Rauhut R, Lendeckel W, Tuschl T (2001). Identification of novel genes coding for small expressed RNAs. Science.

[B4] Aravin AA, Lagos-Quintana M, Yalcin A, Zavolan M, Marks D, Snyder B, Gaasterland T, Meyer J, Tuschl T (2003). The small RNA profile during Drosophila melanogaster development. Dev Cell.

[B5] Lai EC, Tomancak P, Williams RW, Rubin GM (2003). Computational identification of Drosophila microRNA genes. Genome Biol.

[B6] Ruby JR, Jan C, Player C, Axtell MJ, Lee W, Nusbaum C, Ge H, Bartel DP (2006). Large-scale sequencing reveals 21U-RNAs and additional microRNAs and endogenous siRNAs in C. elegans. Cell.

[B7] Berezikov E, Guryev V, van de Belt J, Wienholds E, Plasterk RH, Cuppen E (2005). Phylogenetic shadowing and computational identification of human microRNA genes. Cell.

[B8] Lim LP, Lau NC, Weinstein EG, Abdelhakim A, Yekta S, Rhoades MW, Burge CB, Bartel DP (2003). The microRNAs of Caenorhabditis elegans. Genes Dev.

[B9] Washietl S, Hofacker IL, Lukasser M, Huttenhofer A, Stadler PF (2005). Mapping of conserved RNA secondary structures predicts thousands of functional noncoding RNAs in the human genome. Nat Biotechnol.

[B10] Nam JW, Shin KR, Han J, Lee Y, Kim VN, Zhang BT (2005). Human microRNA prediction through a probabilistic co-learning model of sequence and structure. Nucleic Acids Res.

[B11] Wang X, Zhang J, Li F, Gu J, He T, Zhang X, Li Y (2005). MicroRNA identification based on sequence and structure alignment. Bioinformatics.

[B12] Xue C, Li F, He T, Liu GP, Li Y, Zhang X (2005). Classification of real and pseudo microRNA precursors using local structure-sequence features and support vector machine. BMC Bioinformatics.

[B13] Sewer A, Paul N, Landgraf P, Aravin A, Pfeffer S, Brownstein MJ, Tuschl T, van Nimwegen E, Zavolan M (2005). Identification of clustered microRNAs using an ab initio prediction method. BMC Bioinformatics.

[B14] Hertel J, Stadler PF (2006). Hairpins in a Haystack: recognizing microRNA precursors in comparative genomics data. Bioinformatics.

[B15] Lewis BP, Burge CB, Bartel DP (2005). Conserved seed pairing, often flanked by adenosines, indicates that thousands of human genes are microRNA targets. Cell.

[B16] Grun D, Wang YL, Langenberger D, Gunsalus KC, Rajewsky N (2005). microRNA target predictions across seven Drosophila species and comparison to mammalian targets. PLoS Comput Biol.

[B17] Enright AJ, John B, Gaul U, Tuschl T, Sander C, Marks DS (2003). MicroRNA targets in Drosophila. Genome Biol.

[B18] Lewis BP, Shih IH, Jones-Rhoades MW, Bartel DP, Burge CB (2003). Prediction of mammalian microRNA targets. Cell.

[B19] Krek A, Grun D, Poy MN, Wolf R, Rosenberg L, Epstein EJ, MacMenamin P, da Piedade I, Gunsalus KC, Stoffel M (2005). Combinatorial microRNA target predictions. Nat Genet.

[B20] Lai EC (2002). Micro RNAs are complementary to 3' UTR sequence motifs that mediate negative post-transcriptional regulation. Nat Genet.

[B21] Stark A, Brennecke J, Russell RB, Cohen SM (2003). Identification of Drosophila MicroRNA targets. PLoS Biol.

[B22] Brennecke J, Stark A, Russell RB, Cohen SM (2005). Principles of microRNA-target recognition. PLoS Biol.

[B23] Xie X, Lu J, Kulbokas EJ, Golub TR, Mootha V, Lindblad-Toh K, Lander ES, Kellis M (2005). Systematic discovery of regulatory motifs in human promoters and 3' UTRs by comparison of several mammals. Nature.

[B24] Chan CS, Elemento O, Tavazoie S (2005). Revealing Posttranscriptional Regulatory Elements Through Network-Level Conservation. PLoS Comput Biol.

[B25] Sinha S, Blanchette M, Tompa M (2004). PhyME: a probabilistic algorithm for finding motifs in sets of orthologous sequences. BMC Bioinformatics.

[B26] Gertz J, Riles L, Turnbaugh P, Ho SW, Cohen BA (2005). Discovery, validation, and genetic dissection of transcription factor binding sites by comparative and functional genomics. Genome Res.

[B27] Loots GG, Ovcharenko I, Pachter L, Dubchak I, Rubin EM (2002). rVista for comparative sequence-based discovery of functional transcription factor binding sites. Genome Res.

[B28] Boffelli D, McAuliffe J, Ovcharenko D, Lewis KD, Ovcharenko I, Pachter L, Rubin EM (2003). Phylogenetic shadowing of primate sequences to find functional regions of the human genome. Science.

[B29] Cliften P, Sudarsanam P, Desikan A, Fulton L, Fulton B, Majors J, Waterston R, Cohen BA, Johnston M (2003). Finding functional features in Saccharomyces genomes by phylogenetic footprinting. Science.

[B30] Siepel A, Bejerano G, Pedersen JS, Hinrichs AS, Hou M, Rosenbloom K, Clawson H, Spieth J, Hillier LW, Richards S (2005). Evolutionarily conserved elements in vertebrate, insect, worm, and yeast genomes. Genome Res.

[B31] Elemento O, Tavazoie S (2005). Fast and systematic genome-wide discovery of conserved regulatory elements using a non-alignment based approach. Genome Biol.

[B32] He L, Thomson JM, Hemann MT, Hernando-Monge E, Mu D, Goodson S, Powers S, Cordon-Cardo C, Lowe SW, Hannon GJ, Hammond SM (2005). A microRNA polycistron as a potential human oncogene. Nature.

[B33] Hayashita Y, Osada H, Tatematsu Y, Yamada H, Yanagisawa K, Tomida S, Yatabe Y, Kawahara K, Sekido Y, Takahashi T (2005). A polycistronic microRNA cluster, miR-17-92, is overexpressed in human lung cancers and enhances cell proliferation. Cancer Res.

[B34] Lau NC, Lim LP, Weinstein EG, Bartel DP (2001). An abundant class of tiny RNAs with probable regulatory roles in Caenorhabditis elegans. Science.

[B35] Castillo-Davis CI, Hartl DL (2003). GeneMerge-postgenomic analysis, datamining, and hypothesis testing. Bioinformatics.

[B36] Ashburner M, Ball CA, Blake JA, Botstein D, Butler H, Cherry JM, Davis AP, Dolinski K, Dwight SS, Eppig JT (2000). Gene ontology: tool for the unification of biology. The Gene Ontology Consortium. Nat Genet.

[B37] Camon E, Magrane M, Barrell D, Lee V, Dimmer E, Maslen J, Binns D, Harte N, Lopez R, Apweiler R (2004). The Gene Ontology Annotation (GOA) Database: sharing knowledge in Uniprot with Gene Ontology. Nucleic Acids Res.

[B38] Gene Ontology Consortium (2004). The Gene Ontology(GO) project in 2006. Nucleic Acids Res.

[B39] Stark A, Brennecke J, Bushati N, Russell RB, Cohen SM (2005). Animal MicroRNAs confer robustness to gene expression and have a significant impact on 3'UTR evolution. Cell.

[B40] Karolchik D, Baertsch R, Diekhans M, Furey TS, Hinrichs A, Lu YT, Roskin KM, Schwartz M, Sugnet CW, Thomas DJ (2003). The UCSC Genome Browser Database. Nucleic Acids Res.

[B41] Hinrichs AS, Karolchik D, Baertsch R, Barber GP, Bejerano G, Clawson H, Diekhans M, Furey TS, Harte RA, Hsu F (2006). The UCSC Genome Browser Database: update 2006. Nucleic Acids Res.

[B42] Clote P The Altschul-Erikson algorithm. http://bioinformatics.bc.edu/clotelab/RNAdinucleotideShuffle/dinucleotideShuffle.html.

[B43] Washietl S Alifoldz algorithm. http://www.tbi.univie.ac.at/papers/SUPPLEMENTS/Alifoldz/.

[B44] Griffiths-Jones S, Grocock RJ, van Dongen S, Bateman A, Enright AJ (2006). miRBase: microRNA sequences, targets and gene nomenclature. Nucleic Acids Res.

[B45] Gu J The pairwise conservation score program. http://bioinfo.au.tsinghua.edu.cn/member/~gujin/pcs/.

[B46] Valentini G, Dietterich TG (2003). Low Bias Bagged Support Vector Machines. The Twentieth International Conference on Machine Learning, ICML.

[B47] Chang C, Lin C LIBSVM: a library for support vector machines. http://www.csie.ntu.edu.tw/~cjlin/libsvm.

[B48] Hofacker IL, Fontana W, Stadler PF, Bonhoeffer S, Tacker M, Schuster P (1994). Fast Folding and Comparison of RNA Secondary Structures. Monatshefte f Chemie.

[B49] Crooks GE, Hon G, Chandonia JM, Brenner SE (2004). WebLogo: A sequence logo generator. Genome Res.

[B50] Altschul SF, Madden TL, Schaffer AA, Zhang J, Zhang Z, Miller W, Lipman DJ (1997). Gapped BLAST and PSI-BLAST: a new generation of protein database search programs. Nucleic Acids Res.

