# Supplementary figures and images for: Identifications of conserved 7-mers in 3'-UTRs and microRNAs in Drosophila
Source: BMC Bioinformatics. 2007 Nov 8;8:432. doi: 10.1186/1471-2105-8-432 (PMC2241842; doi:10.1186/1471-2105-8-432)

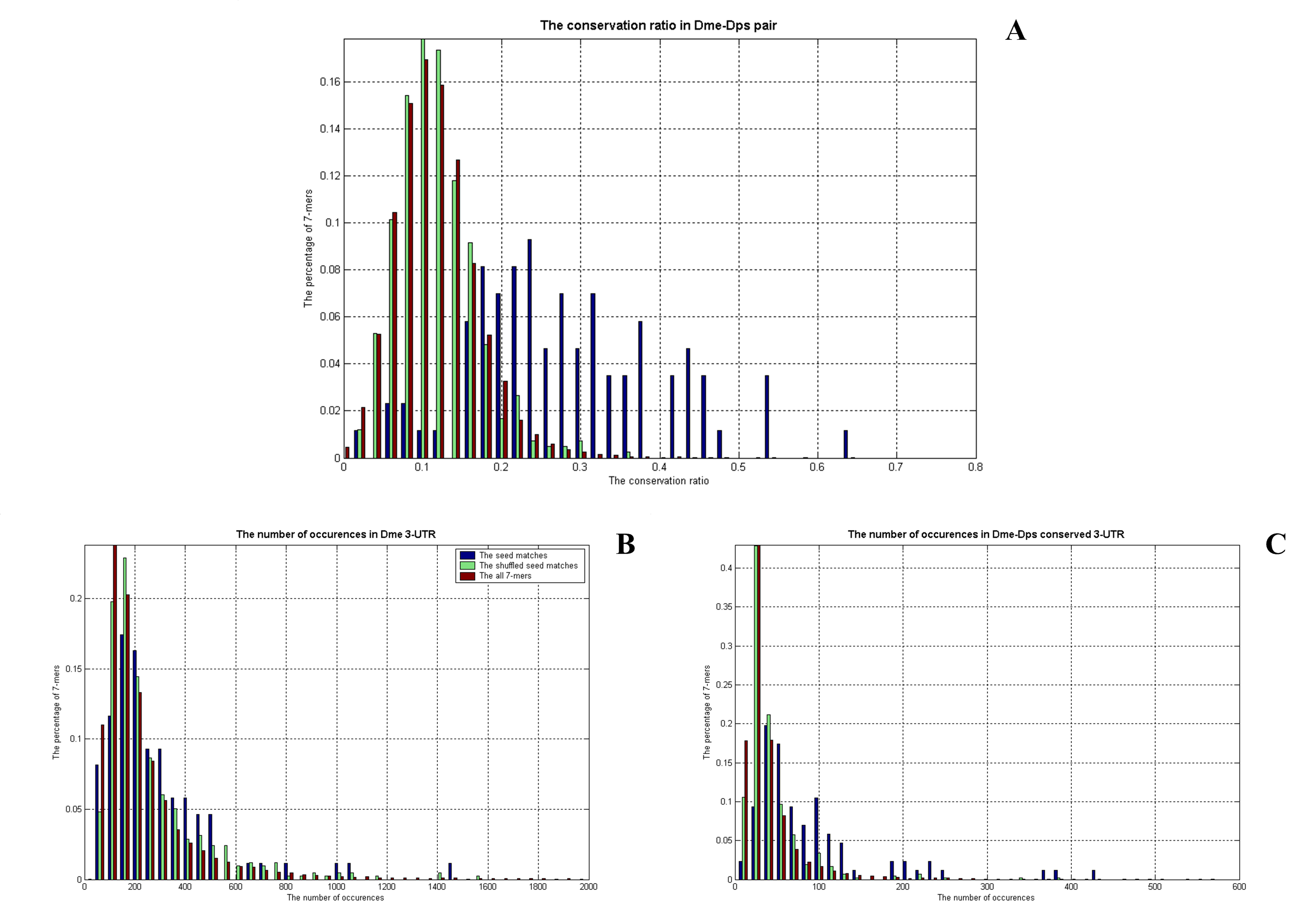

Supplement: Additional file 1 — The distributions of conservation ratios and the counts in Dme-Dps pair. Figure S1. The distributions are computed and plotted for three dataset: reference seed matches, shuffled seed matches and all 7-mers. A) The distribution of conservation ratios. B) The distribution of counts in Dme 3'-UTRs. C) The distribution of counts in Dme-Dps conserved 3'-UTRs. [file 1471-2105-8-432-S1.png]

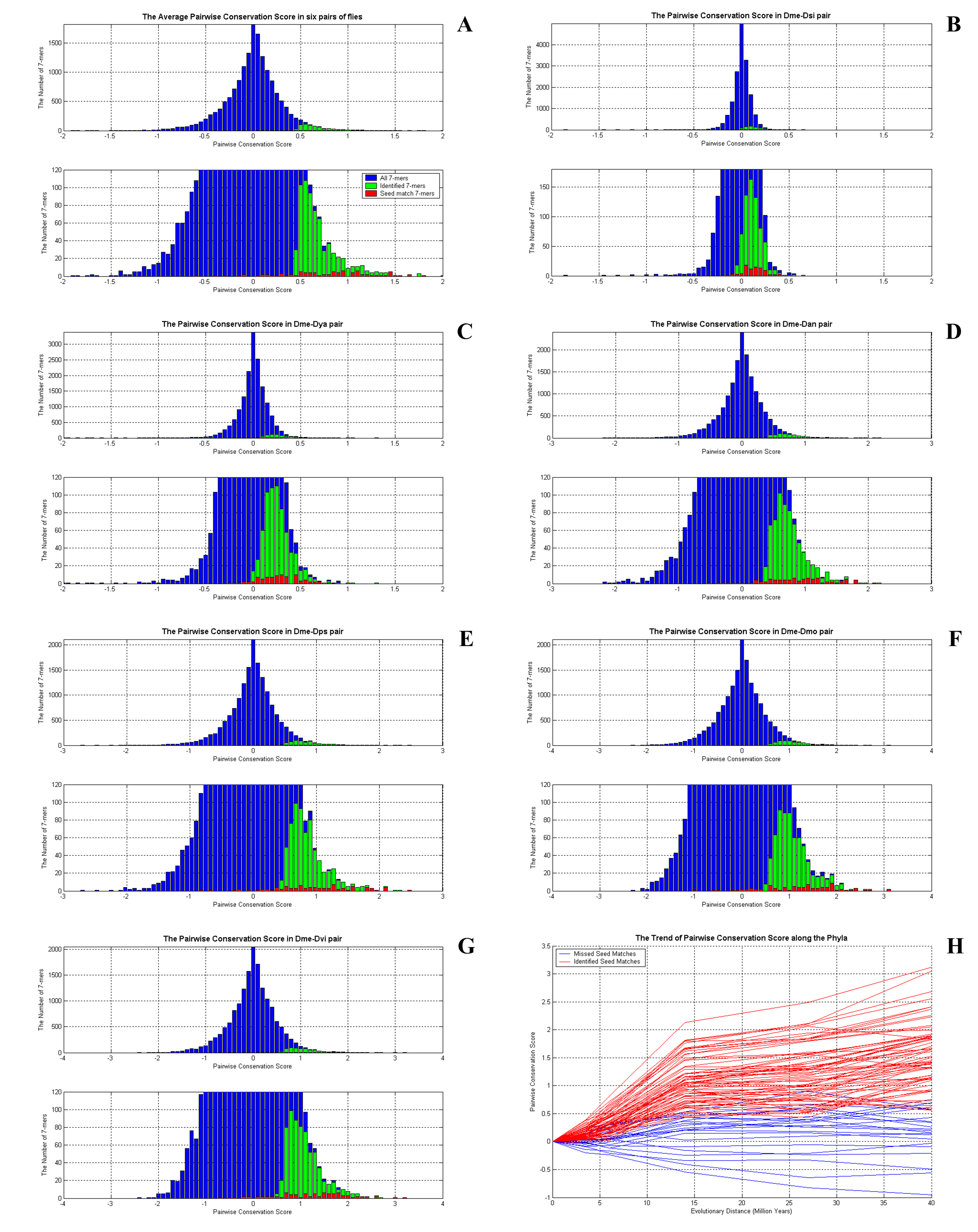

Supplement: Additional file 2 — The histograms of PCSs and the trends of PCSs along the phyla. Figure S2. A)-G) The PCSs of all 7-mers, from left to right: the average PCSs and the PCSs in Dme-Dsi, Dme-Dya, Dme-Dan, Dme-Dps, Dme-Dmo, Dme-Dvi. The top panel of each sub-figure shows the histograms of the PCSs of all the 7-mers and the bottom panel shows the enlarged visions. H) The trends of the PCSs of 86 seed matches along the phyla. Because the evolutionary distances of the Dme-Dmo and Dme-Dvi pairs are the same, only the PCSs of the Dme-Dmo pairs are displayed. [file 1471-2105-8-432-S2.png]

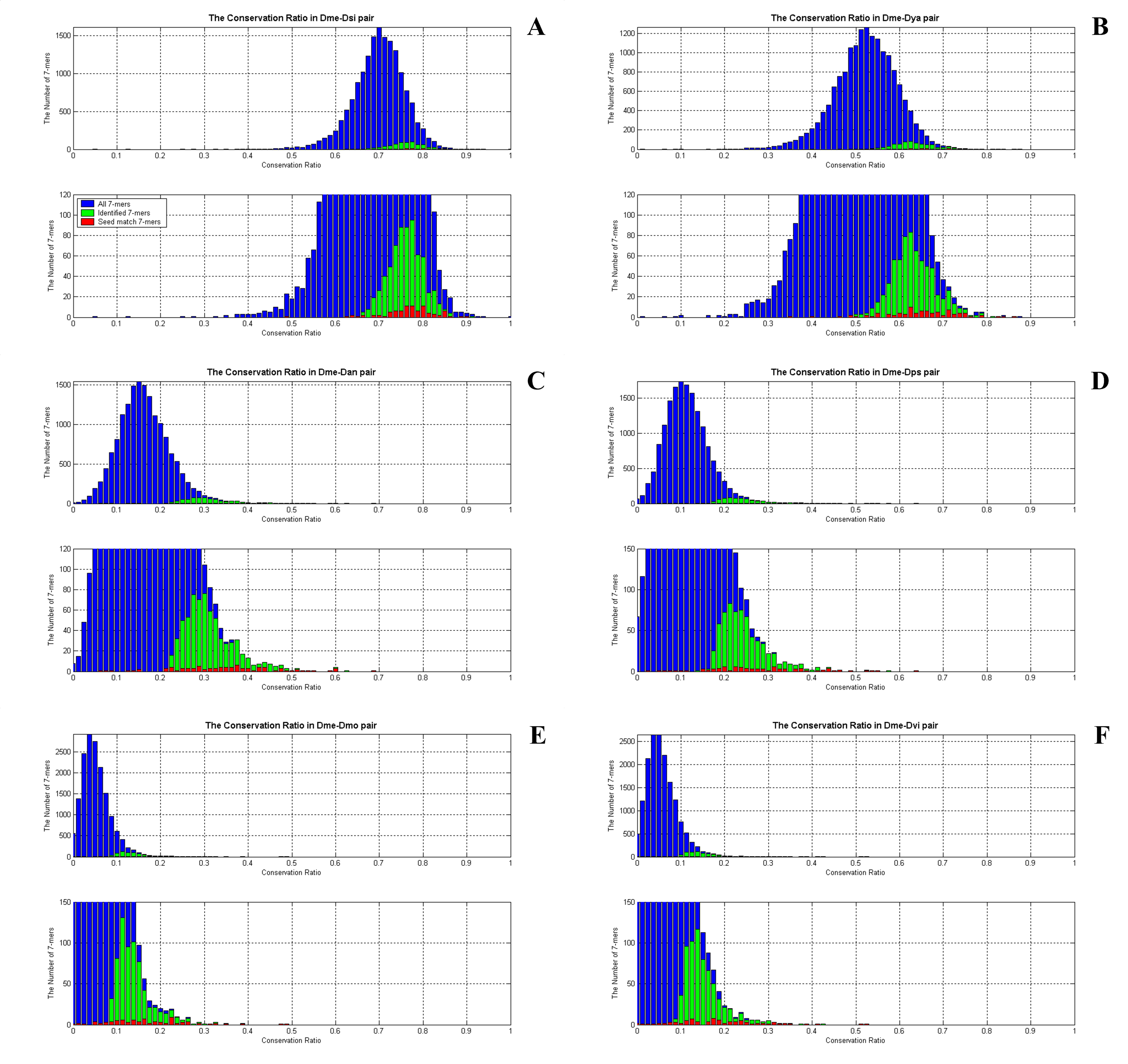

Supplement: Additional file 3 — The histograms of conservation ratios along the phyla. Figure S3. A)-F) The conservation ratios of all 7-mers, from left to right: the conservation ratios in Dme-Dsi, Dme-Dya, Dme-Dan, Dme-Dps, Dme-Dmo, Dme-Dvi. The top panel of each sub-figure shows the histograms of the conservation ratios of all the 7-mers and the bottom panel shows the enlarged visions. [file 1471-2105-8-432-S3.png]
